# Attenuation of porcine deltacoronavirus disease severity by porcine reproductive and respiratory syndrome virus coinfection in a weaning pig model

**DOI:** 10.1080/21505594.2021.1908742

**Published:** 2021-04-02

**Authors:** Xinrong Zhou, Xinna Ge, Yongning Zhang, Jun Han, Xin Guo, Yanhong Chen, Lei Zhou, Hanchun Yang

**Affiliations:** Key Laboratory of Animal Epidemiology of Ministry of Agriculture and Rural Affairs, College of Veterinary Medicine, China Agricultural University, Beijing, P.R. China

**Keywords:** Porcine deltacoronavirus, porcine reproductive and respiratory syndrome virus, coinfection, proinflammatory cytokines, pathogenicity

## Abstract

Porcine deltacoronavirus (PDCoV) is a potentially emerging zoonotic pathogen that causes severe diarrhea in young pigs, with a risk of fatal dehydration. Its pathogenicity on neonatal piglet has been previously reported, however, it is less known if the coinfection with immunosuppressive pathogens can influence PDCoV disease manifestation. Here, a coinfection model of PDCoV and porcine reproductive and respiratory syndrome virus (PRRSV), a global-spread immunosuppressive virus, was set to study their interaction. Weaning pigs in the coinfection group were intranasally inoculated with PRRSV NADC30-like virus and latterly orally inoculated with PDCoV at three day-post-inoculation (DPI). Unexpectedly, compared with pigs in the PDCoV single-infected group, the coinfected pigs did not show any obvious diarrhea, as PDCoV fecal shedding, average daily weight gain (ADWG), gross and microscopic lesions and PDCoV IHC scores consistently indicated that PRRSV coinfection lessened PDCoV caused diarrhea. Additionally, three proinflammatory cytokines TNF-α, IL-1 and IL-6, which can be secreted by PRRSV infected macrophages, were detected to be highly expressed at the intestine from both PRRSV infected groups. By adding to PDCoV-infected cells, these three cytokines were further confirmed to be able to inhibit the PDCoV replication post its cellular entry. Meanwhile, the inhibition effect of the supernatant from PRRSV-infected PAMs could be obviously blocked by the antagonist of these three cytokines. In conclusion, PRRSV coinfection increased TNF-α, IL-1, and IL-6 in the microenvironment of intestines, which inhibits the PDCoV proliferation, leading to lessened severity of diarrhea. The findings provide some new insight into the pathogenesis and replication regulation of PDCoV.

## Introduction

Porcine deltacoronavirus (PDCoV) is a potentially emerging zoonotic pathogen, first identified in Hong Kong in 2012 [1], which has been widely reported from the United States, China, Vietnam, and many other countries worldwide [2–8]. The PDCoV infected pigs can show acute severe watery diarrhea and vomiting that lead to dehydration, bodyweight loss, even potentially fatal consequences [4,5]. PDCoV belongs to the genus coronavirus of the family *Coronaviridae* in the order *Nidovirales*. And it has an enveloped, single-stranded, positive-sense RNA genome, approximately 25.4 kb [1,6–8]. Currently, it has been reported that PDCoV can efficiently infect cells from a broad range of species by using various species’ aminopeptidase N (APN) receptors, including porcine, feline, human, and chicken [9]. Considering many pandemic human coronaviruses, such as severe acute respiratory syndrome (SARS)-CoV, Middle East respiratory syndrome coronavirus (MERS-CoV), have a zoonotic origin, the concerns on PDCoV’s cross-species transmission and zoonotic potentials are raising. Except for infecting intestine tissues and causing diarrhea, PDCoV also shows a wider tropism in non-enteric tissues, compared with other enteric coronaviruses, such as porcine epidemic diarrhea virus (PEDV) [10]. These characters increase the complexity of PDCoV’s pathogenesis.

Porcine reproductive and respiratory syndrome virus (PRRSV) is an economically important pathogen, causing reproductive failure in sows and respiratory dysfunction in all ages of pigs, which has widespread in almost all pork-producing countries [11–13]. PRRSV is also characterized by its immunosuppression, which markedly alters innate immunity, Treg cells activation, inflammatory and immunoregulatory cytokine secretion in different manners [14–16]. For example, PRRSV-infected macrophages can secrete proinflammatory cytokines including TNF‐α, IL‐1, and IL‐6 that play a significant role in the immunoregulatory and antiviral activity [17,18].

On intensive pig farms, the coinfections of PRRSV with other pathogens, such as porcine circovirus type 2 (PCV2), swine influenza virus (SIV), and porcine respiratory coronavirus (PRCV) are very common [19–24]. As a consequence, coinfection can usually induce more severe clinical signs and lung lesions than those associated with infection by either agent alone, suggesting that the coinfection of PRRSV and other respiratory pathogens is generally considered to have a synergistic effect. Besides, PRRSV infection can disturb the host immune response against CSFV vaccinations as well [23,25]. So, coinfection and superinfection are potentially serious threats to public health and animal husbandry, which may lead to more serious and chronic diseases when secondary/opportunistic pathogens invaded. However, if PRRSV infection can increase the severity of PDCoV-mediated disease or even change its symptom pattern has not been reported yet. To study the interaction between PDCoV and PRRSV, a weaning pig model of coinfection was established here. By comparing the clinical symptom, viral shedding, gross and microscopic lesions, and IHC scores between single- and coinfected pigs, we found that PRRSV infection 3 days before PDCoV inoculation can lessen the severity of PDCoV-mediated diarrhea. And the cytokines TNF-α, IL-1, and IL-6 in the microenvironment of the intestines induced by PRRSV infection, were further identified as the contributors to inhibit PDCoV replication. The findings provided some new insight into the pathogenesis and replication regulation of PDCoV.

## Materials and methods

### Cells and viruses

For proliferation and titration, MA104 derived monkey kidney cells, i.e., MARC-145 and porcine intestinal epithelium cell lines, i.e., IPEC-J2, for PRRSV and PDCoV, respectively, were cultured in DMEM (Thermo Fisher Scientific) supplemented with 10% fetal bovine serum (FBS) (Gibco). Porcine alveolar macrophages (PAMs), the target cells of PRRSV, were prepared from 38-day-old specific-pathogen-free (SPF) pigs and grown in the RPMI 1640 medium (Thermo Fisher Scientific) with 10% FBS, as previously described [26]. All the cells were cultured at 37 °C in a humid 5% CO_2_ incubator.

A Chinese PDCoV strain CHN-HN-1601 (GenBank accession no. MG832584) [27] and a moderate pathogenic PRRSV strain CHsx1401 (GenBank accession no. KP861625) [28], representing the current predominant Lineage 1 virus in both China and the United States [11,29], were used at the 8^th^ passage, as previous study [26].

### Indirect immunofluorescence assay (IFA)

Paraformaldehyde (4%) fixed cells were incubated at 37 °C for 1 h with monoclonal antibodies (mAb) SDOW-17 (RTI) (against PRRSV) [30] or 1A3 (against PDCoV) made by our lab [27]. Alexa-fluor-488-conjugated goat anti-mouse polyclonal antibody (dilution 1:1000) (Invitrogen) was used as the secondary antibody. The images were taken with a fluorescence microscope (ECLIPSE Ci-S) [31].

### Virus titration

As per the previous description, the titers of PRRSV and PDCoV were tested on the MARC-145 and IPEC-J2 cells with IFA, respectively, and measured as 50% tissue culture infective dose per mL (TCID_50_/mL) according to the Reed–Muench method [32,33].

### Western blot (WB)

Total 2 μg protein from NP-40 lysed cells was separated by SDS-PAGE and then transferred to a PVDF membrane (Millipore). After blocking with 5% (w/v) skimmed-milk, the membranes were incubated with specific mAb against PDCoV N protein and horseradish peroxidase (HRP)-conjugated goat anti-mouse antibodies (dilution 1:5000) (Abcam) as primary and secondary antibodies, respectively. Membranes were treated with enhanced chemiluminescence (ECL) (Pierce) [34] and signals were detected by chemiluminescence apparatus (ProteinSimple).

### Animal study

To reduce the interference from other swine pathogens, 24 18-day-old landrace pigs, free of PRRSV, CSFV, PRV, PCV2, Mycoplasma hyopneumoniae, PDCoV, PEDV, and TGEV were purchased from Beijing Center for SPF Swine Breeding & Management and raised in the facility of China Agricultural University (CAU). The pigs were randomly divided into four groups (n = 6): PDCoV, PRRSV, PDCoV + PRRSV coinfection, and mock group. The process of inoculation was shown in Table 1. Rectal temperatures and clinical signs, such as fever, cough, rhinorrhea, and diarrhea were recorded daily and scored as in our previous study [35]. Both nasal and rectal swabs were collected daily to monitor the virus shedding. At 0, 7, 14, and 21 DPI the sera were collected and the body weights of the pigs were recorded. Once severe diarrhea was observed, three pigs in each group were randomly selected for euthanasia and necropsy. The gross lesions of the lungs and intestines were observed and scored as the previous description [27,35]. The lung and intestine samples were fixed for histopathological and immunohistochemistry examination. The rest of the pigs were euthanized for necropsy at 21 DPI, following the same process.

**Table 1.** Programs for inoculation and sample collection in animal studies

DPI/Groups	PDCoV	PDCoV+PRRSV	PRRSV	Mock
0 DPI^@^	SC* and BW*, IN* DMEM 2 mL	SC and BW, IN PRRSV^#^ 2 mL	SC and BW, IN PRRSV 2 mL	SC and BW, IN DMEM 2 mL
3 DPI	OR* PDCoV^#^ 2 mL	OR PDCoV 2 mL	OR DMEM 2 mL	OR DMEM 2 mL
7 DPI	SC and BW,	SC and BW,	SC and BW,	SC and BW,
8 DPI	Euthanized 3 pigs	Euthanized 3 pigs	Euthanized 3 pigs	Euthanized 3 pigs
14 DPI	SC and BW	SC and BW	SC and BW	SC and BW
21 DPI	SC and BW, Euthanized 3 pigs	SC and BW, Euthanized 3 pigs	SC and BW, Euthanized 3 pigs	SC and BW, Euthanized 3 pigs

@ Rectal temperatures and clinical signs such as fever, cough, rhinorrhea, and diarrhea were daily recorded and scored.

* SC means serum collection; IN means intranasal inoculation; BW means body weighting; OR means oral inoculation.

# PRRSV: CHsx1401 strain (1 × 10^5^ TCID_50_/mL); PDCoV: CHN-HN-1601 strain (1 × 10^8^ TCID_50_/mL).

### Real-time RT-PCR for PDCoV and PRRSV quantification

RT-qPCR primers (PDCoV: forward 5´-AGGGTTCGGGAGCTGACACTTCT-3´, reverse 5´-GGTCGCGTTTCCTGGGCTGATT-3´; PRRSV: forward 5´-ATGATGRGCTGGCATTCT-3´, reverse 5´-ACACGGTCGCCCTAATTG-3´) were designed and the viral RNA load in each sample was tested as previous description [27,32].

### Histopathology and immunohistochemistry (IHC)

Lung and intestine tissues were fixed and processed by routine histopathological and IHC procedures as previous [30]. For IHC, PRRSV and PDCoV in the lungs and intestines samples were stained with mAb SDOW-17 and 1A3 (1: 2000 dilution), respectively, and HRP labeled goat-anti-mouse secondary antibody (Abcam). And the scores were evaluated based on the severity of lesions and the distribution of viral antigen in each section as previously described [28].

### Detecting the production levels of TNF-α, IL-1, and IL-6 in intestines and supernatants of PRRSV infected PAMs

As PRRSV infection could induce a large number of proinflammatory secretion, which might be related to the inhibition of secondary virus replication. In this study, the production level of TNF-α, IL-1, and IL-6 in the intestinal microenvironment was detected. 1 g ileum tissues collected from inoculated pig were ground with 1 mL PBS. After a centrifuge at 4 °C 12,000 × g for 10 min, the TNF-α, IL-1, and IL-6 in the supernatants were tested using ELISA kits (CUSABIO BIOTECH). Correspondently, these three cytokines in the supernatant of PRRSV (MOI = 0.1) infected PAMs at 24 hpi were also tested using the same kits.

### Inhibition effects of PRRSV-infected PAMs supernatants and TNF-α, IL-1, and IL-6 on PDCoV proliferation

To evaluate if the increased TNF-α, IL-1, and IL-6 in the intestinal microenvironment contribute to the inhibition of PDCoV proliferation, the inhibition effects of PRRSV-infected PAMs’ supernatants and the three cytokines on PDCoV proliferation were explored *in vitro*. 200 µl supernatants collected from PRRSV-infected PAMs at 24 hpi was mixed with 300 µl DMEM diluted PDCoV and added to IPEC-J2 cells (MOI = 0.1) in the 6-well cell culture plates for 2 h incubation. After 3 times washing with PBS, 2 mL medium including 5 μg/mL trypsin and 500 µl PRRSV-infected PAMs supernatants was added and then the viral titer and protein expression level of PDCoV were detected at 24 hpi. The supernatants from non-infected PAMs and DMEM were set as the mock and negative controls.

Similarly, the inhibition effect of TNF-α, IL-1, and IL-6 (Abcam) with the concentration up to 20 ng/mL, 40 ng/mL, and 40 ng/mL, according to the detected concentration in the intestines, were also tested, respectively. To further confirm the inhibition effect of these three cytokines, their inhibitors adalimumab (TNF-α inhibitor, Sigma), anakinra (IL-1 receptor antagonist, MedChemExpress), and tocilizumab (IL-6 receptor neutralizing antibody, MedChemExpress) with two different concentrations (1 µg/mL or 10 µg/mL) were added into the PRRSV-infected PAMs supernatants to detected if they can block the inhibition effect. The viral titers of PDCoV were detected at 24 hpi as well.

The cell viability post the treatment of cytokines or their inhibitors was measured by cell counting kit-8 (Beyotime biotechnology).

### Determine the inhibition stages of PDCoV proliferation

To identify the inhibition stages of TNF-α, IL-1, and IL-6 on the PDCoV proliferation, cytokine mixtures (20 ng/mL TNF-α, 40 ng/mL IL-1, and 40 ng/mL IL-6) and medium diluted PDCoV (MOI = 0.1) were added to the IPEC-J2 cells in the 6-well plates (500 µL/well). After 2 h absorption at 37 °C in the 5% CO_2_ incubator, the medium was removed, followed by 3 times PBS wash; then, a batch of inoculated cells was harvested within 200 μl/well PBS using the cell scraper. Parallelly, the rest batch of cells was further cultured to 12 hpi in the medium with the same concentration of cytokines and 5 µg/mL trypsin. The harvested viruses were quantitated by WB and RT-qPCR to evaluate the inhibition effect of these three cytokines on virus attachment/entry and replication. The PDCoV infected IPEC-J2 cells without cytokine treatment were set as control.

### Ethics statement

All protocols for PAMs preparation and animal inoculation were approved by the Laboratory Animal Ethical Committee of CAU, with approval No. AW01049102-2-2.

### Statistical analysis

The data in animal trials or *in vitro* test were analyzed using two-way ANOVA in the software GraphPad Prism (version 5.0). The data were expressed as means ± standard deviations (SD). Differences were considered statistically significant at a *p*-value of <0.05.

## Results

### PRRSV inoculation 3 days before PDCoV inoculation lessens the severity of diarrhea in weaning pig

**Figure 1.**
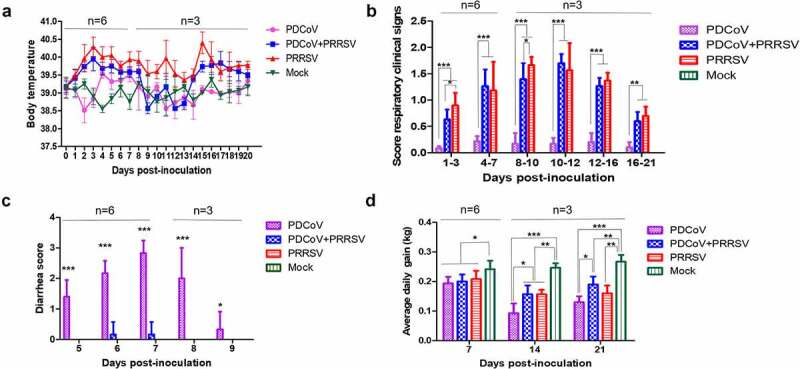
Clinical assessment of inoculated pigs. The body temperatures, average respiratory and diarrhea clinical scores and ADWG of inoculated pigs were shown as means ± standard deviations (error bars), the animal amount n = 6 at 0 to 8 DPI and n = 3 at 9 to 21 DPI. The asterisk indicates a significant difference between labeled groups (*p < 0.05; **p < 0.01; ***p < 0.001). (a) The rectal temperature of pigs was daily recorded and shown. (b) Respiratory related clinical signs including cough, fever, anorexia, and sneeze, were scored from 0 to 20, and the mean values of the labeled period were calculated. (c) The diarrhea was daily scored and the mean values of each group at 5 to 9 DPI were individually shown. (d) The ADWG was calculated at 7, 14 and 21 DPI

To evaluate the influence of PRRSV coinfection on PDCoV disease severity in weaning pigs, 18-day-old SPF pigs were inoculated with PRRSV 3 days before PDCoV inoculation, and two single inoculation groups and mock were set, respectively. The average body temperature of PRRSV infected pigs in both groups raised at 3 DPI and reached above 40 °C at 6 DPI-10 DPI and 12 DPI-15 DPI, with the highest temperature of 40.7 °C and 40.5 °C in the PRRSV single-infection and coinfection groups, respectively (Figure 1(a)). As expected, all pigs in these two groups showed characteristic signs including coughing, sneezing, labored breathing, and anorexia from 3 DPI onwards. The respiratory-related symptoms scores of both PRRSV infection groups are similar to each other, except at the 1–3 and 8–10 DPI, but they are significantly higher than that of the other two groups (*p* < 0.01) (Figure 1(b)). Meanwhile, fever and respiratory symptoms were not observed in PDCoV single-infection and mock groups. For diarrhea, clinical signs mostly appeared in the PDCoV single-infected pigs at 5–9 DPI, with a diarrhea rate of 4/6 and the highest diarrhea score around 3. Surprisingly, only one coinfected pig had soft feces at 6–7 DPI, and the diarrhea scores were less than 1 (Figure 1(c)). ADWG of the PDCoV single-infection group was 0.09 kg and 0.13 kg at 14 DPI and 21 DPI, gaining only half of the mock, which were also significantly lower than that in the coinfection group (*p* < 0.05) (Figure 1(d)). These data indicated that PRRSV infection 3 days before PDCoV lessened the severity of diarrhea in weaned pigs, which was completely contrary to our original expectation.

### PRRSV coinfection reduces the viral shedding of PDCoV

**Figure 2.**
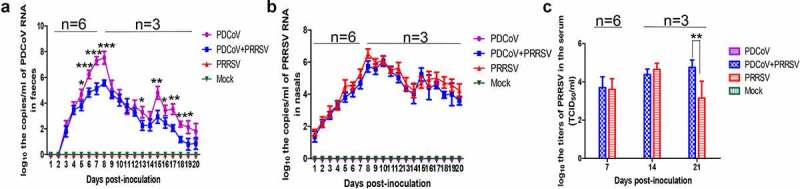
Viral shedding and viremia. The viral RNA shedding kinetics of PDCoV in fecal swab (a) and PRRSV in nasal swab (b) were tested by RT-qPCR and the titers of PRRSV viremia (c) were test by TCID_50_ assay, at 7, 14 and 21 DPI. Data were shown as means ± standard deviations (error bars), the animal amount n = 6 at 0 to 8 DPI and n = 3 at 9 to 21 DPI. The asterisk indicates a significant difference between PDCoV single-infection and coinfection groups (*p < 0.05; **p < 0.01; ***p < 0.001)

The rectal and nasal swabs were collected daily to detect the viral shedding by RT-qPCR. PDCoV shedding could be initially detected at 4 DPI and reached the peak around 8 DPI in both PDCoV-inoculated groups, and the average copy number of PDCoV in the single-infection group could reach 10^7.53 copies/mL, 100 times higher than that of the coinfection group at the virus shedding peak. And the average copy numbers of the PDCoV single-infected group were significantly higher than that of the coinfection group at 5–8, 13, and 15–19 DPI (*p* < 0.01 or *p* < 0.05). There was no PDCoV detected in the PRRSV single-infection group and mock (Figure 2(a)). Meanwhile, both PRRSV inoculated groups showed similar viral shedding pattern in the whole test period and no PRRSV shedding was detected in the other two groups.

### Viremia

Sera collected at 7, 14, and 21 DPI were submitted to detect the viral titers. Same as our previous study, the PDCoV viremia was undetectable in sera. And PRRSV titers were similar between single-infection and coinfection groups at 7 and 14 DPI, and their peak titers were close, reaching 10^^^4.65 TCID_50_/mL at 14 DPI in the single-infection group and 10^^^4.75 TCID_50_/mL at 21 DPI in the coinfection group (Figure 2(c)).

### Gross pathology, histopathology and IHC results indicate lessened severity of PDCoV-mediated disease in co-infected pigs

**Figure 3.**
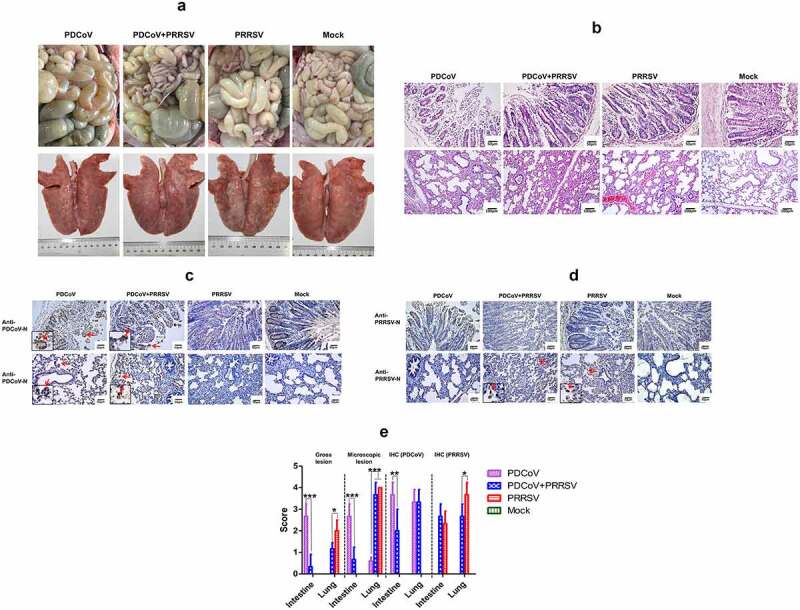
Gross and microscopic lesions and IHC examination of inoculated pigs. 3 pigs from each group were randomly selected for necropsy at 8 DPI, for observing the gross and microscopic lesions and virus distribution. (a) Shown are the representative pictures of intestines and lungs gross lesions. (b) Shown are the representative pictures of intestines (up panel) and lungs (down panel) microscopic pathological lesions. Intestines and lung sections were stained with hematoxylin and eosin (H & E). (c) PDCoV and (d) PRRSV distribution in intestines (up panel) and lungs (down panel) were shown in IHC examination, stained by mAbs against their N protein, respectively. Red arrows indicate the positive signals (stained in brown) in virus-infected cells. (e) The scores of gross and microscopic lesions, PDCoV and PRRSV distribution in both intestines and lungs were shown. Data were shown as means ± standard deviations (error bars) (n = 3). The asterisk indicates a significant difference between labeled groups (*p < 0.05; **p < 0.01; ***p < 0.001)

After diarrhea was observed in the inoculated pigs, three pigs from each group were randomly selected for necropsy at 8 DPI, and the rest of the pigs were necropsied at 21 DPI. Yellow, soft to watery contents in the small intestines, thin-walled and/or gas-distended small intestines, and gas-distended colons were observed in the PDCoV single-infection group at 8 DPI. However, only very slight lesions were found in the coinfection group, and there was no obvious intestine lesion observed in the PRRSV single-infection group and mock. At 21 DPI, there was already no noticeable intestine lesion observed among all groups. For lung lesions, the typical PRRSV-mediated pneumonia was shown in both PRRSV-inoculated groups, with similar severity. And only a few minor interstitial pneumonia was found in PDCoV single-infected pigs (Figure 3(a)).

Throughout the small intestine section, mild to severe villous atrophy was mainly present in PDCoV single-infected pigs, shown as multifocal to diffuse villous enterocyte swelling and vacuolation, as well as moderate to severe villous blunting and atrophy. Only one pig in the coinfection group showed similar microscopic lesions, but it was very slight (Figure 3(b)). The lesion scores from HE stained slices were consistent with the gross lesions.

By immunohistochemistry (IHC), PDCoV antigen could be detected in the cytoplasm of villous enterocytes in pigs in both PDCoV-infected groups. IHC scores based on the ratio of virus-infected cells further suggest that the PRRSV pre-infection suppresses PDCoV replication *in vivo* (Figure 3(c, d)). No PDCoV antigen was detected in the sections of the mock.

### PRRSV infection increases the concentration of TNF-α, IL-1, and IL-6 in the microenvironment of intestines and PAMs supernatant

**Figure 4.**
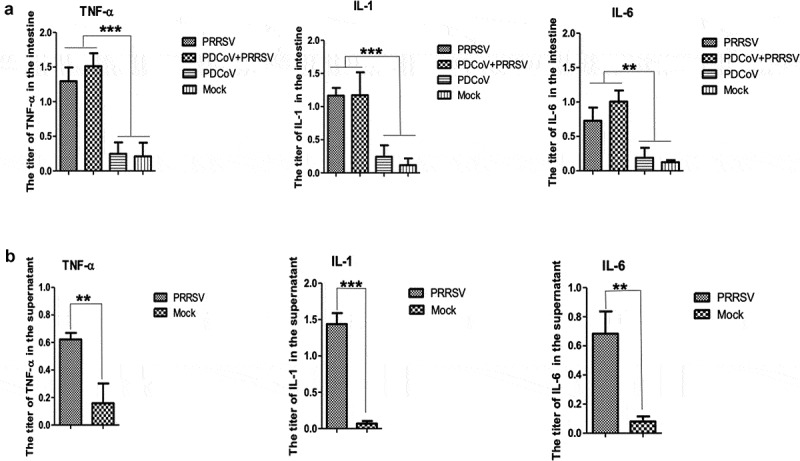
Detecting TNF-α, IL-1, and IL-6 in intestines and supernatants of PRRSV infected PAMs. Shown are titers of cytokines TNF-α, IL-1, and IL-6 in intestines (a) at 8 DPI and in the supernatants of PRRSV infected PAMs at 24 hpi (b), detected by ELISA kits. Data were shown as means ± standard deviations (error bars). The asterisk indicates a significant difference between labeled groups (*p < 0.05; **p < 0.01; ***p < 0.001)

It was reported that PRRSV infection could highly induce PAMs secreting cytokines TNF-α, IL-1, and IL-6. To confirm if they contribute to inhibiting PDCoV proliferation, these three cytokines in the microenvironment of the intestines were quantified. The results showed that their titers were significantly increased to almost 4–6 times in both PRRSV-infected groups compared with PDCoV single-infected group and mock (Figure 4(a)). Meanwhile, the secretion of these three cytokines in the supernatant of PRRSV infected PAMs was also confirmed, with the concentrations ranged from 5 to 40 ng/mL, calculated according to the established standard curve of the kits (Figure 4(b)).

### TNF-α, IL-1 and IL-6 inhibit the proliferation of PDCoV in the IPEC-J2 cells

**Figure 5.**
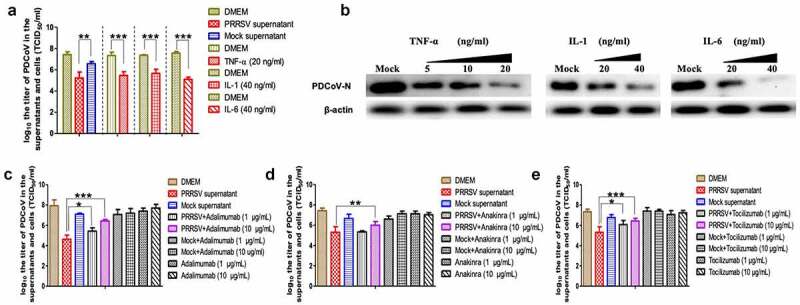
The inhibiting effect of PRRSV-infected PAMs supernatants and TNF-α, IL-1, and IL-6 on PDCoV proliferation in IPEC-J2 cells. (a) The titers of PDCoV in the IPEC-J2 cells treated with PRRSV-infected PAMs supernatants, TNF-α, IL-1, and IL-6, respectively. (b) The expression levels of N protein in the PDCoV-infected IPEC-J2 cells treated with different concentrations of TNF-α, IL-1, and IL-6 were measured by WB. (c), (d), and (e) The titers of PDCoV in the IPEC-J2 cells treated with PRRSV-infected PAMs supernatants together with TNF-α, IL-1, and IL-6 inhibitors, respectively. (*p<0.05; **p<0.01; ***p<0.001)

To testify the hypothesis that these three cytokines could inhibit the proliferation of PDCoV, the supernatants of PRRSV-infected PAMs and the recombinant TNF-α (20 ng/mL), IL-1 (40 ng/mL) and IL-6 (40 ng/mL) were added into the culture medium of PDCoV-infected IPEC-J2 cells, and then their effects on the viral replication were evaluated via detecting viral titer and WB. The results demonstrated that the supernatant of PRRSV-infected PAMs and theses three cytokines could suppress PDCoV replication (Figure 5(a, b)). To further confirm the specificity of these cytokines suppressing PDCoV replication, their inhibitors were added to the supernatants of PRRSV-infected PAMs and viral loads of PDCoV significantly increased in Figure 5(c–e) suggesting that these three cytokines could inhibit the PDCoV proliferation and it might play an important role in the phenomenon.

### TNF-α, IL-1, and IL-6 inhibit the proliferation of PDCoV at the replication stage

**Figure 6.**
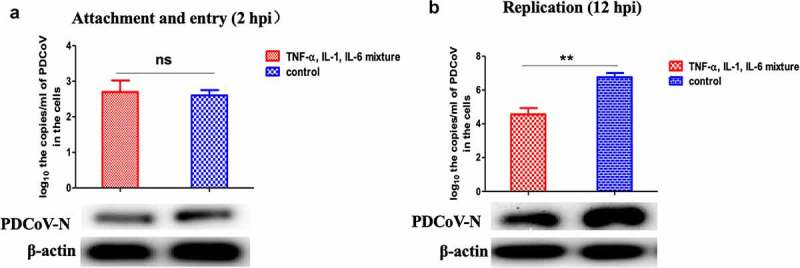
Determine the inhibition stages of PDCoV proliferation by TNF-α, IL-1, and IL-6 mixtures. Cells were harvested at 2 hpi and 12 hpi, respectively, for RT-qPCR and WB analysis, after treating with TNF-α, IL-1, and IL-6 mixtures. The results showed that there was no difference at the attachment/entry stage and significant difference appeared at the replication stage, suggesting that TNF-α, IL-1, and IL-6 mixtures inhibited PDCoV proliferation at the replication stage

To further determine the stage that TNF-α, IL-1, and IL-6 inhibit the PDCoV proliferation, the efficiency of viral attachment/entry and replication in the cytokines treated IPEC-J2 cells, were evaluated by RT-qPCR and WB at 2 and 12 hpi. The results suggest that there is no significant difference at the attachment/entry (Figure 6(a)) but the cytokines could inhibit the PDCoV replication after entry (Figure 6(b)).

## Discussion

In the early reports, PDCoV pathogenicity was mainly evaluated through field observation or inoculation experiments with neonatal piglets [4,10]. For PDCoV positive farm, coinfection with multiple pathogens is very common [36], so it is well concerned if the coinfection with the immunosuppressive virus could impact PDCoV pathogenesis, leading to more severity of the disease. Considering its inter-species transmission possibility and potential zoonotic risk, it is significant to address the mechanism of PDCoV pathogenicity under different conditions and pig ages. Meanwhile, PRRSV has been widely reported to suppress the host immune system, increasing the susceptibility and severity to coinfection or secondary infections of other pathogens [18], and decreasing the vaccine efficiency [30]. However, how PRRSV affects the disease outcome of enteric coronaviruses is rarely reported. At the beginning, PRRSV NADC30-like strain CHsx1401 picked for this study has been carefully evaluated through multiple aspects, such as its moderate pathogenicity and representative for the currently predominant lineage 1 virus in both China and the United States. In contrast, the highly pathogenic PRRSV strain with fatal virulence was not suitable for analyzing the interaction, as it causes severe pathological lesions and even acute death can easily cover the real influence of PDCoV [12]. Additionally, our previous animal studies showed that CHsx1401-inoculated pigs exhibited obvious clinical signs from 3 DPI onwards; meanwhile, the suckling pigs around 21 days old are still susceptible to PDCoV effectively [5], so the animal age and inoculation process were set as above to investigate if PRRSV coinfection could increase the severity of the disease, even cause mortality in non-neonatal pigs.

Unexpectedly, the lessened symptoms, together with lighter enteric lesions, lower PDCoV shielding and reduced positive signals in enteric samples tested by IHC, were observed in the coinfection group. These data indicated that PRRSV coinfection significantly inhibited the proliferation of PDCoV *in vivo*. Referring to previous studies described [37,38], viral interference was a common phenomenon in superinfection or coinfection and the mechanism underlying this phenomenon is generally related to the interference in the stage of secondary virus attachment, entry, genome replication, and budding. Hence, to further explore this mechanism, we initially have investigated the susceptibility of IPEC-J2 cells and PAMs to both PRRSV and PDCoV, but failed to coinfect either kind of cells *in vitro*. Additionally, according to IHC staining results, there was no obvious PRRSV signal found in the enteric epithelial cells and the major target cells of PDCoV, indicating that PRRSV and PDCoV do not interact in the coinfected cells by sharing cell tropism. It is well known that PRRSV has a restricted cell tropism and prefers to invade well-differentiated cells of the monocyte/macrophage lineage [39], and IPEC-J2 cells might have no suitable receptors for PRRSV attachment and entry or the intracellular environment of IPEC-J2 cells does not support PRRSV replication. So PRRSV should take other pathways to interrupt PDCoV proliferation.

We surmised that several reasons might be related. Firstly, as the previous studies have reported, low pathogenic PRRSV-like CHsx1401 enhanced the production of pro-inflammatory cytokines including IL-1, IL-6 and TNF-α in PAMs and serum [40], which could inhibit infection of other pathogens through the activation of NF-КB pathway or STAT pathway [34]. Besides, upon the PRRSV infection, GLUT2 mRNA abundance and sucrase, maltase, and Na+/K+ adenosine triphosphatase activities in the intestines had increased, and the changed intestinal microenvironment might suppress PDCoV infection in the epithelial cells [41,42]. Additionally, PRRSV infection might inhibit the secretion of trypsin based on the fact that PRRSV infection could induce the occurrence of pancreatitis [43] and the reduced trypsin might down-regulate the expression levels of receptors for PDCoV entry. In this study, we mainly focused on testifying if the proinflammatory cytokines had the inhibited effects on the PDCoV replication.

Cytokines are cell-signaling agents that can coordinate innate and adaptive immune responses against pathogens. Among them, type I interferons (IFN) is well characterized for inducing antiviral responses; however, PRRSV can significantly interfere with its synthesis and secretion in the infected PAMs [40], so it was not analyzed in this study. However, the proinflammatory cytokines are well induced in PRRSV infected PAMs, including TNF‐α, IL‐1, and IL‐6 [37], which have been reported to be able to suppress the CSFV replication through activating NF-кB or STAT pathway [34]. To confirm the speculation if these cytokines might also contribute to PDCoV inhibition, their expression level in intestines tissues of different groups and the supernatants from PRRSV infected PAMs were first quantified. Notably, TNF‐α, IL‐1, and IL‐6 were not greatly induced in the intestine of the PDCoV single infection group and mock, whose level was significantly lower than that in both PRRSV infected groups. This was consistent with the previous report about cytokine expression level in Peyer’s patches of PDCoV inoculated pigs [44]. And then their inhibition effect was investigated *in vitro* with similar concentrations detected *in vivo*. The results showed that the PRRSV-induced TNF‐α, IL‐1, and IL‐6 could inhibit PDCoV replication effectively in a dose-dependent way. And most importantly, the inhibition effect of PAMs’ supernatant could be specifically blocked by the antagonist of TNF‐α, IL‐1, and IL‐6, respectively, through a dose-dependent way. These data provide some solid evidence to support that TNF‐α, IL‐1, and IL‐6 contribute to the PDCoV inhibition, even though, here we cannot completely rule out the roles played by other cytokines yet.

TNF-α is a powerful proinflammatory agent involved in the innate immune response and ít mediates the release of various cytokines from stimulated macrophages, such as IL-1 and IL-6. Meanwhile, it can activate multiple antiviral pathways and synergize with IFN-γ in promoting antiviral activities, which has been widely reported in influenza virus, vesicular stomatitis virus, and encephalomyocarditis virus [45–47]. In addition, previous mechanistic studies revealed that IL-1 can induce an IFN-like state that restricts viral replication [48]. And IL-6 is a pleiotropic cytokine that participate in infection, playing a role in antiviral effect through activating the JAK-STAT signaling pathway [49–51]. However, it still needs to further explore the mechanism of how proinflammatory cytokines suppress the proliferation of PDCoV and how PDCoV interacts with them to affect the disease outcome. This will be a very interesting project for our future research.

In summary, by using weaning pigs, we successfully set up a PRRSV and PDCoV coinfection model and found that PRRSV infection 3 days before PDCoV can lessen the severity of PDCoV-mediated diarrhea and reduce the pathological lesions, PDCoV tissue distribution, and viral shielding. Furthermore, we confirmed that PRRSV infection-induced TNF-α, IL-1, and IL-6 in the microenvironment of intestines, contribute to the inhibition of PDCoV replication. This founding dose not only provides some new insight into the pathogenesis and replication regulation of PDCoV but it also gives us a clue for antiviral strategy development in the future.

## Supplementary Material

Supplemental Material

## References

[1] Woo PC, Lau SK, Lam CS, et al. Discovery of seven novel Mammalian and avian coronaviruses in the genus deltacoronavirus supports bat coronaviruses as the gene source of alphacoronavirus and betacoronavirus and avian coronaviruses as the gene source of gammacoronavirus and deltacoronavirus. J Virol. 2012;86(7):3995–4008. 22278237 10.1128/JVI.06540-11 PMC3302495

[2] Huang H, Yin Y, Wang W, et al. Emergence of Thailand-like strains of porcine deltacoronavirus in Guangxi Province, China. Vet Med Sci. 2020;6(4):854–859. 10.1002/vms3.283 PMC7738719 32419393

[3] Sun W, Wang L, Huang H, et al. Genetic characterization and phylogenetic analysis of porcine deltacoronavirus (PDCoV) in Shandong Province, China. Virus Res. 2020;278:197869. 31962065 10.1016/j.virusres.2020.197869 PMC7114949

[4] Chen Q, Gauger P, Stafne M, et al. Pathogenicity and pathogenesis of a United States porcine deltacoronavirus cell culture isolate in 5-day-old neonatal piglets. Virology. 2015;482:51–59. 25817405 10.1016/j.virol.2015.03.024 PMC7111688

[5] Dong N, Fang L, Yang H, et al. Isolation, genomic characterization, and pathogenicity of a Chinese porcine deltacoronavirus strain CHN-HN-2014. Vet Microbiol. 2016;196:98–106. 27939164 10.1016/j.vetmic.2016.10.022 PMC7117368

[6] Hu H, Jung K, Vlasova AN, et al. Isolation and characterization of porcine deltacoronavirus from pigs with diarrhoea in the United States. J Clin Microbiol. 2015;53(5):1537–1548. 25740769 10.1128/JCM.00031-15 PMC4400786

[7] Song D, Zhou X, Peng Q, et al. Newly emerged porcine deltacoronavirus associated with diarrhoea in swine in China: identification, prevalence and full-length genome sequence analysis. Transbound Emerg Dis. 2015;62(6):575–580. 26250097 10.1111/tbed.12399 PMC7169704

[8] Wang L, Byrum B, Zhang Y. Detection and genetic characterization of delta coronavirus in pigs, Ohio, USA, 2014. Emerg Infect Dis. 2014;20(7):1227–1230. 24964136 10.3201/eid2007.140296 PMC4073853

[9] Li W, Hulswit RJG, Kenney SP, et al. Broad receptor engagement of an emerging global coronavirus may potentiate its diverse cross-species transmissibility. Proc Natl Acad Sci U S A. 2018;115(22):E5135–E5143. 29760102 10.1073/pnas.1802879115 PMC5984533

[10] Hu H, Jung K, Vlasova AN, et al. Experimental infection of gnotobiotic pigs with the cell-culture-adapted porcine deltacoronavirus strain OH-FD22. Arch Virol. 2016;161(12):3421–3434. 27619798 10.1007/s00705-016-3056-8 PMC7087098

[11] Jiang Y, Li G, Yu L, et al. Genetic diversity of porcine reproductive and respiratory syndrome virus (PRRSV) from 1996 to 2017 in China. Front Microbiol. 2020;11:618. 32390968 10.3389/fmicb.2020.00618 PMC7193098

[12] Zhou L, Zhang J, Zeng J, et al. The 30-amino-acid deletion in the nsp2 of highly pathogenic porcine reproductive and respiratory syndrome virus emerging in China is not related to its virulence. J Virol. 2009;83(10):5156–5167. 19244318 10.1128/JVI.02678-08 PMC2682102

[13] Kappes MA, Faaberg KS. PRRSV structure, replication and recombination: origin of phenotype and genotype diversity. Virology. 2015;479-480:475–486. 25759097 10.1016/j.virol.2015.02.012 PMC7111637

[14] Butler JE, Lager KM, Golde W, et al. Porcine reproductive and respiratory syndrome (PRRS): an immune dysregulation pandemic. Immunol Res. 2014;59(1–3):81–108. 24981123 10.1007/s12026-014-8549-5 PMC7091131

[15] Lunney JK, Fang Y, Ladinig A, et al. Porcine reproductive and respiratory syndrome virus (PRRSV): pathogenesis and interaction with the immune system. Annu Rev Anim Biosci. 2016;4:129–154. 26646630 10.1146/annurev-animal-022114-111025

[16] Huang C, Zhang Q, Feng WH. Regulation and evasion of antiviral immune responses by porcine reproductive and respiratory syndrome virus. Virus Res. 2015;202:101–111. 25529442 10.1016/j.virusres.2014.12.014 PMC7132515

[17] Liu Y, Shi W, Zhou E, et al. Dynamic changes in inflammatory cytokines in pigs infected with highly pathogenic porcine reproductive and respiratory syndrome virus. Clin Vaccine Immunol. 2010;17(9):1439–1445. 20631336 10.1128/CVI.00517-09 PMC2944458

[18] Han J, Zhou L, Ge X, et al. Pathogenesis and control of the Chinese highly pathogenic porcine reproductive and respiratory syndrome virus. Vet Microbiol. 2017;209:30–47. 28292547 10.1016/j.vetmic.2017.02.020

[19] Cecere TE, Meng XJ, Pelzer K, et al. coinfection of porcine dendritic cells with porcine circovirus type 2a (PCV2a) and genotype II porcine reproductive and respiratory syndrome virus (PRRSV) induces CD4(+)CD25(+)FoxP3(+) T cells in vitro. Vet Microbiol. 2012;160(1–2):233–239. 22633482 10.1016/j.vetmic.2012.04.040 PMC3443269

[20] Jimenez LF, Ramirez Nieto G, Alfonso VV, et al. Association of swine influenza H1N1 pandemic virus (SIV-H1N1p) with porcine respiratory disease complex in sows from commercial pig farms in Colombia. Virol Sin. 2014;29(4):242–249. 25160760 10.1007/s12250-014-3471-5 PMC7091121

[21] Galina L, Pijoan C, Sitjar M, et al. Interaction between streptococcus suis serotype 2 and porcine reproductive and respiratory syndrome virus in specific pathogen-free piglets. Vet Rec. 1994;134(3):60–64. 8135015 10.1136/vr.134.3.60

[22] Lim SI, Jeoung HY, Kim B, et al. Impact of porcine reproductive and respiratory syndrome virus and porcine circovirus-2 infection on the potency of the classical swine fever vaccine (LOM strain). Vet Microbiol. 2016;193:36–41. 27599928 10.1016/j.vetmic.2016.07.027

[23] Wang X, Mu G, Dang R, et al. Up-regulation of IL-10 upon PRRSV vaccination impacts on the immune response against CSFV. Vet Microbiol. 2016;197:68–71. 27938685 10.1016/j.vetmic.2016.11.007

[24] De Bruin MG, Samsom JN, Voermans JJ, et al. Effects of a porcine reproductive and respiratory syndrome virus infection on the development of the immune response against pseudorabies virus. Vet Immunol Immunopathol. 2000;76(1–2):125–135. 10973691 10.1016/S0165-2427(00)00208-7 PMC7119737

[25] Li H, Yang H. Infection of porcine reproductive and respiratory syndrome virus suppresses the antibody response to classical swine fever virus vaccination. Vet Microbiol. 2003;95(4):295–301. 12935755 10.1016/s0378-1135(03)00158-5

[26] Zhang H, Guo X, Ge X, et al. Changes in the cellular proteins of pulmonary alveolar macrophage infected with porcine reproductive and respiratory syndrome virus by proteomics analysis. J Proteome Res. 2009;8(6):3091–3097. 19341299 10.1021/pr900002f

[27] Zhou X, Zhou L, Zhang P, et al. A strain of porcine deltacoronavirus: genomic characterization, pathogenicity and its full-length cDNA infectious clone. Transbound Emerg Dis. 2020. 10.1111/tbed.13862 33012120

[28] Zhou L, Wang Z, Ding Y, et al. NADC30-like strain of porcine reproductive and respiratory syndrome virus, China. Emerg Infect Dis. 2015;21(12):2256–2257. 26584305 10.3201/eid2112.150360 PMC4672414

[29] Paploski IAD, Corzo C, Rovira A, et al. Temporal dynamics of co-circulating lineages of porcine reproductive and respiratory syndrome virus. Front Microbiol. 2019;10:2486. 31736919 10.3389/fmicb.2019.02486 PMC6839445

[30] Zhou L, Yang B, Xu L, et al. Efficacy evaluation of three modified-live virus vaccines against a strain of porcine reproductive and respiratory syndrome virus NADC30-like. Vet Microbiol. 2017;207:108–116. 28757009 10.1016/j.vetmic.2017.05.031

[31] Li Y, Zhou L, Zhang J, et al. Nsp9 and nsp10 contribute to the fatal virulence of highly pathogenic porcine reproductive and respiratory syndrome virus emerging in China. PLoS Pathog. 2014;10(7):e1004216. 24992286 10.1371/journal.ppat.1004216 PMC4081738

[32] Du J, Ge X, Liu Y, et al. Targeting swine leukocyte antigen class I molecules for proteasomal degradation by the nsp1alpha replicase protein of the Chinese highly pathogenic porcine reproductive and respiratory syndrome virus strain JXwn06. J Virol. 2016;90(2):682–693. 26491168 10.1128/JVI.02307-15 PMC4702659

[33] LaBarre DD, Lowy RJ. Improvements in methods for calculating virus titer estimates from TCID50 and plaque assays. J Virol Methods. 2001;96(2):107–126. 11445142 10.1016/s0166-0934(01)00316-0

[34] Chen D, Liu X, Xu S, et al. TNF-alpha induced by porcine reproductive and respiratory syndrome virus inhibits the replication of classical swine fever virus C-strain. Vet Microbiol. 2019;234:25–33. 31213269 10.1016/j.vetmic.2019.05.007

[35] Halbur PG, Paul PS, Frey M, et al. Comparison of the antigen distribution of two US porcine reproductive and respiratory syndrome virus isolates with that of the Lelystad virus. Vet Pathol. 1996;33(2):159–170. 8801709 10.1177/030098589603300205

[36] Zhang F, Luo S, Gu J, et al. Prevalence and phylogenetic analysis of porcine diarrhoea associated viruses in southern China from 2012 to 2018. BMC Vet Res. 2019;15(1):470. 31881873 10.1186/s12917-019-2212-2 PMC6935106

[37] Kumar N, Sharma S, Barua S, et al. Virological and immunological outcomes of coinfections. Clin Microbiol Rev. 2018;31(4):e00111–17. 29976554 10.1128/CMR.00111-17 PMC6148187

[38] Kumar N, Barua S, Riyesh T, et al. Complexities in isolation and purification of multiple viruses from mixed viral infections: viral interference, persistence and exclusion. PLoS One. 2016;11(5):e0156110. 27227480 10.1371/journal.pone.0156110 PMC4881941

[39] Morgan SB, Frossard JP, Pallares FJ, et al. Pathology and virus distribution in the lung and lymphoid tissues of pigs experimentally inoculated with three distinct type 1 PRRS virus isolates of varying pathogenicity. Transbound Emerg Dis. 2016;63(3):285–295. 25382098 10.1111/tbed.12272

[40] Zhang L, Liu J, Bai J, et al. Comparative expression of Toll-like receptors and inflammatory cytokines in pigs infected with different virulent porcine reproductive and respiratory syndrome virus isolates. Virol J. 2013;10:135. 23631691 10.1186/1743-422X-10-135 PMC3673858

[41] An T, Li J, Su C, et al. Molecular and cellular mechanisms for PRRSV pathogenesis and host response to infection. Virus Res. 2020;286:197980. 32311386 10.1016/j.virusres.2020.197980 PMC7165118

[42] Schweer WP, Pearce SC, Burrough ER, et al. The effect of porcine reproductive and respiratory syndrome virus and porcine epidemic diarrhea virus challenge on growing pigs II: intestinal integrity and function. J Anim Sci. 2016;94(2):523–532. 27065122 10.2527/jas.2015-9836

[43] Xu L, Zhou L, Sun W, et al. Nonstructural protein 9 residues 586 and 592 are critical sites in determining the replication efficiency and fatal virulence of the Chinese highly pathogenic. Virology. 2018;517:135–147. 29397202 10.1016/j.virol.2018.01.018 PMC7111471

[44] Xu Z, Zhong H, Huang S, et al. Porcine deltacoronavirus induces TLR3, IL-12, IFN-alpha, IFN-beta and PKR mRNA expression in infected Peyer’s patches in vivo. Vet Microbiol. 2019;228:226–233. 30593372 10.1016/j.vetmic.2018.12.012 PMC7117130

[45] Seo SH, Webster RG. Tumor necrosis factor alpha exerts powerful anti-influenza virus effects in lung epithelial cells. J Virol. 2002;76:1071–1076. 11773383 10.1128/JVI.76.3.1071-1076.2002 PMC135862

[46] Ruby J, Bluethmann H, Peschon JJ. Antiviral activity of tumor necrosis factor (TNF) is mediated via p55 and p75 TNF receptors. J Exp Med. 1997;186(9):1591–1596. 9348317 10.1084/jem.186.9.1591 PMC2199110

[47] Park YK, Park ES, Kim DH, et al. Cleaved c-FLIP mediates the antiviral effect of TNF-alpha against hepatitis B virus by dysregulating hepatocyte nuclear factors. J Hepatol. 2016;64(2):268–277. 26409214 10.1016/j.jhep.2015.09.012

[48] Orzalli MH, Smith A, Jurado KA, et al. An antiviral branch of the IL-1 signaling pathway restricts immune-evasive virus replication. Mol Cell. 2018;71:825–840. 30100266 10.1016/j.molcel.2018.07.009 PMC6411291

[49] Heinrich PC, Behrmann I, Muller-Newen G, et al. Interleukin-6-type cytokine signalling through the gp130/Jak/STAT pathway. Biochem J. 1998;334(Pt 2)(Pt 2):297–314. 9716487 10.1042/bj3340297 PMC1219691

[50] Hunter CA, Jones SA. IL-6 as a keystone cytokine in health and disease. Nat Immunol. 2015;16(5):448–457. 25898198 10.1038/ni.3153

[51] Kim HY, Eo EY, Park H, et al. Medicinal herbal extracts of sophorae radix, acanthopanacis cortex, sanguisorbae radix and torilis Fructus inhibit coronavirus replication in vitro. Antivir Ther. 2010;15(5):697–709. 20710051 10.3851/IMP1615

