# Supplementary material for: Attenuation of porcine deltacoronavirus disease severity by porcine reproductive and respiratory syndrome virus coinfection in a weaning pig model
Source: Virulence. 2021 Apr 2;12(1):1011–21. doi: 10.1080/21505594.2021.1908742 (PMC8023240; doi:10.1080/21505594.2021.1908742)
Supplement: Supplemental Material [file KVIR_A_1908742_SM8258.zip › Document.rtf]

Supplementary figure
Identify the susceptibility of PAMs and IPEC-J2 cells to PRRSV and PDCoV. To investigate whether IPEC-J2 and PAM cells are suitable for PRRSV and PDCoV coinfection, they were individually inoculated with these two viruses (MOI=0.1) respectively. At 24 hpi, the infectivity of PRRSV and PDCoV were detected by IFA. The results suggested neither IPEC-J2 nor PAMs is suitable for testing the coinfection of PDCoV and PRRSV in vitro.
