# Supplementary figures and images for: Attenuation of porcine deltacoronavirus disease severity by porcine reproductive and respiratory syndrome virus coinfection in a weaning pig model
Source: Virulence. 2021 Apr 2;12(1):1011–21. doi: 10.1080/21505594.2021.1908742 (PMC8023240; doi:10.1080/21505594.2021.1908742)

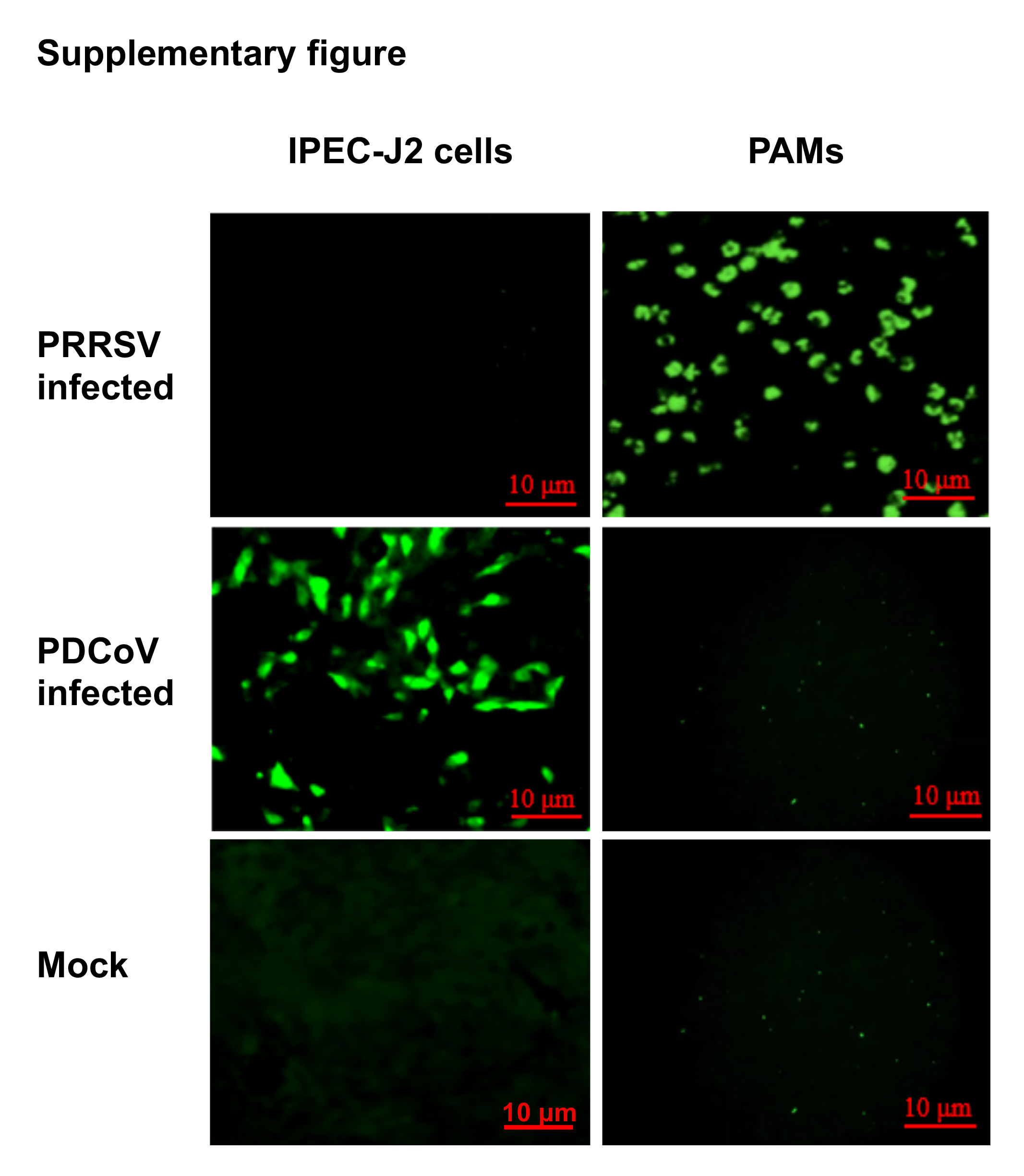

Supplement: Supplemental Material [file KVIR_A_1908742_SM8258.zip › Supplementary figure.tiff]
